# 4169 The influence of serious mental illness on medical care of patients with lower back pain in the emergency department

**DOI:** 10.1017/cts.2020.360

**Published:** 2020-07-29

**Authors:** Courtney Lee, Ian McNeil, Sylvia Guillory, Stacyann Bailey

**Affiliations:** 1 Mount Sinai School of Medicine

## Abstract

OBJECTIVES/GOALS: To determine whether length of stay (LOS) and opioid prescribing differ among patients who present to the emergency department (ED) with low back pain (LBP) and serious mental illness (SMI+) compared to patients without SMI (SMI−). METHODS/STUDY POPULATION: Eligible patients that visited the ED within the Mount Sinai Health Care System from 2016-2019 were identified from the Mount Sinai Data Warehouse. Data on patient demographics, number of medications prescribed, and length of stay (LOS) were compared between the groups. Patients were excluded if English was not their primary language and if the LOS exceeded 24 hours. The final dataset consisted of 940 patients (SMI+: n = 181; SMI−: n = 759). RESULTS/ANTICIPATED RESULTS: SMI+ cases included patients with a diagnosis of depression (n = 152), anxiety (n = 134), schizophrenia (n = 9), bipolar (n = 1), and/or post-traumatic stress disorder (n = 33); 26% of cases had a single diagnosis, 66% with two, and the remaining 8% had three diagnoses. There was no significant difference in pain scores between the two groups (SMI-: 7.0 ± 0.1; SMI+: 6.8 ± 0.3; *p* = 0.6). We found no significant differences in LOS between the groups (SMI-: 3.9 ± 0.1 hours; SMI+: 3.8 ± 0.2 hours; *p* = 0.8), nor was there a significant difference in number of medications prescribed (SMI-: 1.7 ± 0.9; SMI+: 1.7 ± 0.6; *p* = 0.4). Further analysis revealed that the odds of receiving an opiate prescription in the SMI- group was 0.92 (95% CI: 0.54,1.55). DISCUSSION/SIGNIFICANCE OF IMPACT: Comparable opioid prescribing and LOS exist in patients with and without serious mental illness who are seeking treatment for low back pain in the ED. Despite similarities in approaches to care, more information is needed to determine if other social determinants influence these practices.

